# Neural evidence for description dependent reward processing in the framing effect

**DOI:** 10.3389/fnins.2014.00056

**Published:** 2014-03-27

**Authors:** Rongjun Yu, Ping Zhang

**Affiliations:** Department of Psychology and Center for Studies of Psychological Application, School of Psychology, South China Normal UniversityGuangzhou, China

**Keywords:** framing effect, reward, ACC, ERP, FRN

## Abstract

Human decision making can be influenced by emotionally valenced contexts, known as the framing effect. We used event-related brain potentials to investigate how framing influences the encoding of reward. We found that the feedback related negativity (FRN), which indexes the “worse than expected” negative prediction error in the anterior cingulate cortex (ACC), was more negative for the negative frame than for the positive frame in the win domain. Consistent with previous findings that the FRN is not sensitive to “better than expected” positive prediction error, the FRN did not differentiate the positive and negative frame in the loss domain. Our results provide neural evidence that the description invariance principle which states that reward representation and decision making are not influenced by how options are presented is violated in the framing effect.

## Introduction

People make decisions based on their mental representations of problems or options. Theories of rational choice argue that the same option will be evaluated in the same way, regardless of how the option is described; thus, equivalent descriptions should lead to identical decisions, known as the description invariance principle. For example, in the expected utility theory, choice options are strictly evaluated as a function of probability and magnitude with no specification of how probability and magnitude are described (Neumann and Morgenstern, 1947). However, both in real-world situations and in the laboratory, there are many cases that violate the description invariance principle. The same situation may be seen in different ways depending on how it is described. As commonly known, the glass can be described as half full or half empty and be perceived as either positive or negative. Studies have shown that beef described as “75% lean” was given higher ratings and tasted better than beef described as “25% fat” (Levin and Gaeth, 1988). Teams are allocated more funds when their performance rates are framed in terms of successes rather than failures (Duchon et al., 1989).

In the famous Asian disease problem, participants have to choose between two programs to combat the disease. It has been shown that if the description is in terms of affectively positive aspects, that is, the option is the prospect of saving 200 lives out of 600 lives (safe option) vs. saving 600 people with one-third probability and no people with two-third probability (risky option), then risk aversion usually results; if the description is in terms of affectively negative aspects, namely, the option is 400 out of 600 will die and with one-third probability nobody will die vs. with two-third probability 600 people will die, then risk seeking ensues (Tversky and Kahneman, 1981; Kühberger and Gradl, 2011). These two logically equivalent but descriptively different frames led to substantial differences in people's choices. Thus, rating of a single option is reversed depending on the valence emphasis of outcome description even though those presentations are logically equivalent, termed the “framing effect” (Tversky and Kahneman, 1981).

Prospect Theory accounts for the framing effect as a consequence of the decision-maker coding positive descriptions as gains and negative descriptions as losses, inducing risk averse and risk seeking preferences respectively (Tversky and Kahneman, 1981). It predicts that subjective evaluations of options are changed based on descriptions. However, preference reversal at the behavioral level does not always imply description invariance violation. Some argue that the framing simply influences individuals' reasoning process and the construal of the logical content of the problem. For example, the Fuzzy-Trace Theory (FTT) proposes that rather than processing quantitative data, decision makers develop a qualitative representation of the problem (Reyna and Brainerd, 1991; Reyna and Ellis, 1994). In the Asian Disease problem, options may be constructed as: Program A is “Some people will be saved;” Program B is “Some people will be saved or no one will be saved;” Program C is “Some people will die” and Program D is “Nobody will die or some people will die.” Given that “Some people will be saved” is common to A and B, the unique “… no one will be saved” in B shifts preference to A; on the other hand, with C and D, the “… some people will die” is common while the unique “Nobody will die” shifts preference to D. According to the FTT, description invariance is not necessarily violated. Because decision making operates on simplified rather than on exact numerical information, the framing effect occurs (Kuhberger and Tanner, 2010).

Whether frames influence initial option evaluation or subsequent reasoning or both is still an open question. A seminal study using functional magnetic resonance imaging (fMRI) revealed that activity in the amygdala was enhanced when subjects chose in accordance with the frame effect, whereas enhanced activity in the anterior cingulate cortex (ACC) was observed when subjects' choices ran counter to their general behavioral tendency (De Martino et al., 2006). Since a large number of studies have shown that individuals are risk seeking in wins and risk aversion in losses, the general behavioral tendency would be more likely to choose sure options in the positive domain and more likely to choose gamble options in the negative domain, whereas running counter to such tendency would be making opposite choices. Thus, the interaction between decision and frame (Positive_sure—Negative_gamble)—(Positive_gamble—Negative sure) represents the general behavioral tendency, i.e., framing effect (De Martino et al., 2006). These findings suggest that frames influence the decision-making stage. However, it is still unknown whether and how the initial evaluation of stimuli is also influenced by frames.

Our study focuses on the initial option evaluation using high temporal resolution event related brain potentials (ERP). Previous studies have shown that reward processing is very rapid, at around 250 ms after stimulus presentation (Miltner et al., 1997; Gehring and Willoughby, 2002; Holroyd and Coles, 2002; Yeung and Sanfey, 2004; Goyer et al., 2008). It is important to know whether framing influences early stage rapid reward processing or it only influences later stage reward processing. In the early stage, only the most important aspects of outcome, such as valence and magnitude, are processed (Toyomaki and Murohashi, 2005; Holroyd et al., 2006). In the late stage, more contextual information, such as the value relative to expectation and social comparison, are integrated (Peterburs et al., 2013). Behavioral studies show that even after participants are aware of the framing effect, they still show robust framing bias (Schick, 1992), suggesting that the framing effect might happen automatically at a very early stage.

Using ERP, we examine how positive and negative frames are evaluated in both the win and the loss domain. Here, domain means the valence of reward, e.g., wins or losses, whereas frames represent how the advantage of the option (positive) or the disadvantage of the option (negative) is emphasized in the description. It has been emphasized that findings obtained studying preferences in the domain of gains should not be immediately generalized to the domain of losses (Tymula et al., 2013). For example, on average people are much more risk- and ambiguity-tolerant in losses than in gains and these preferences are not correlated with each other (Zhou and Wu, 2011; Tymula et al., 2012, 2013). Individuals were more sensitive to unfairness in the loss domain than in the win domain (Zhou and Wu, 2011; Wu et al., 2014). Thus, it is important to study the neural mechanisms underlying framing effect in both domains.

The feedback related negativity (FRN), an ERP component that peaks at around 250 ms after feedback onset, has been found to be sensitive to the valence of outcomes, being more pronounced for negative feedback associated with unfavorable outcome, such as incorrect response or monetary loss, than for positive feedback (Miltner et al., 1997; Gehring and Willoughby, 2002). The FRN is maximal at frontal-central scalp electrode sites and is generally believed to be generated at the ACC (Miltner et al., 1997; Gehring and Willoughby, 2002). One theory suggests that the FRN reflects the processes of assessing the motivational/affective impact of outcome events, i.e., the processes of putting subjective values onto outcomes (Gehring and Willoughby, 2002; Yeung and Sanfey, 2004). Another influential hypothesis posits that the FRN is elicited by the negative reward prediction error (i.e., “the result is worse than expected”) (Holroyd and Coles, 2002). The negative reward prediction error signal, which is associated with decreased dopaminergic neurons activity, disinhibits ACC neurons, thereby producing the cortical error signal (Nieuwenhuis et al., 2004). Recent studies have shown that the FRN is not only sensitive to monetary outcomes (Dunning and Hajcak, 2007; Bellebaum et al., 2010; Yu et al., 2011). It has been shown that the FRN could be elicited by predictive cues presented prior to feedbacks (Dunning and Hajcak, 2007; Yu et al., 2011). Other studies found that the FRN encodes unfair proposals compared to fair proposals (Boksem and De Cremer, 2010; Campanha et al., 2011; Pfabigan et al., 2011; Osinsky et al., 2013). We predicted that the FRN would be more negative for the negative frames than for the positive frames in the win domain. Since the FRN is more sensitive to “worse than expected” negative prediction error than to “better than expected” positive prediction error, we predicted that the influence of framing on FRN would be significant in the win domain but not in the loss domain. In the loss domain, there is little room to be “worse than expected” since losses are already the worst outcomes. The P300, which is the most positive peak in the 200–600 ms time window post-onset of feedback, has also been shown to be sensitive to the valence of reward (Hajcak et al., 2005; Wu and Zhou, 2009). We predicted that the P300 would be more positive for the positive frame than the negative frame and be more positive for wins than losses.

**Figure 1:**
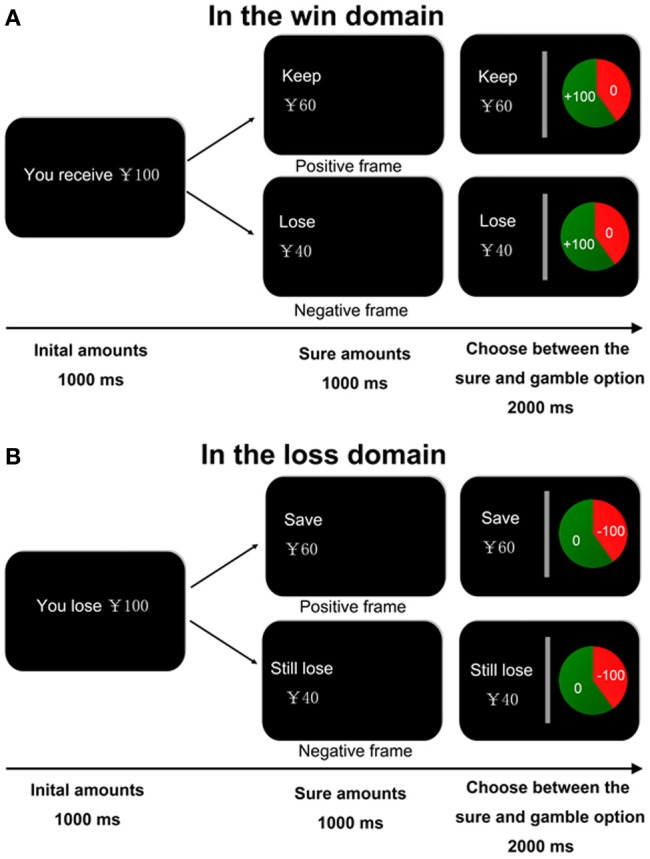
**Experimental task design**. At the beginning of each trial, a message indicating the starting amount of money was presented for 1000 ms. The amount is a win in the win domain **(A)** and a loss in the loss domain **(B)**. The participants were informed that they may or may not receive this initial amount of money, depending on their subsequent choices. Then a sure option was showed in the left of the screen for 1000 ms. The sure option was framed either positive or negative in both domains. After that, a gamble option, that is, a pie chart depicting the probability of winning (i.e., keep all initial reward in the win domain or lose nothing in the loss domain) or losing (i.e., receive nothing in the win domain or lose the initial amount in the loss domain) was showed in the right of the screen for 2000 ms. Participants are required to make a decision within the 2000 ms.

To investigate the effects of framing on reward processing in the human brain, we conducted the experiment with the electroencephalogram (EEG) being recorded when participants performed the framing task (see Figure 1). In Experiment 1, we confirmed that framing influences decision making in both the win and the loss domain, when wins and losses trials are separated into different block. In order to replicate our findings and to further investigate possible interaction between domains and frames, in Experiment 2, we examined these effects when win and loss trials were mixed.

## Materials and methods

### Participants

Eighteen undergraduate students (11 males; mean age ± *SD*, 20.4 ± 1.3 years) participated in Experiment 1 and 14 undergraduate students (7 males; mean age ± *SD*, 21.7 ± 2.2 years) participated in Experiment 2. All the participants were right- handed and had normal or corrected-to-normal vision, and were screened for neurological or psychiatric disorders. The study was approved by the Academic Committee of the School of Psychology at South China Normal University. All participants gave written, informed consent and were informed of their right to discontinue participation at any time.

### Experimental paradigm

Before the experiment, the subjects were familiarized with the decision-making task, and given ten practice trials.

At the beginning of each trial, participants were shown a message (for 1000 ms) indicating the starting amount of money that they would receive. Subjects were instructed that they would not be able to retain the whole of this initial amount, but would next have to choose between a sure and a gamble option. Then the sure option was presented for 1000 ms. In the win block, the sure amount was framed as the amount participants would keep of the initial endowment in the positive frame condition (“
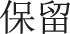
” in Chinese) or as the amount participants would lose of the initial endowment in the negative frame (“
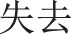
” in Chinese). In the loss block, the sure amount was framed as the amount participants would save in the positive frame (“
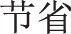
” in Chinese) or as the amount participants would still lose in the negative frame (“
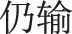
” in Chinese). Participants were then asked to choose between a sure option and a gamble option presented in the context of two different frames. The gamble option was shown as a pie chart depicting the probability of winning or losing in each trial. Participants in the win domain block were told that they would gain all initial money if they won the gamble and would gain nothing if they lost the gamble. In the loss domain block, they would lose no money if they won the gamble and would lose the entire amount at stake if they lost the gamble. The two alternatives were presented in succession. The sure option was presented on the left side of the screen and the gamble option was presented on the right side of the screen. Participants were given 2 s to respond by pressing the left or right button. They were also told that during the task they would not receive feedback concerning the outcomes of their decisions (see Figure 1).

They would be awarded or penalized according to their decision at the end of the experiment. Four different starting amounts were used in the experiment (￥25, ￥50, ￥75, and ￥100). There were four different probabilities of winning or losing in a given trial (20, 40, 60, and 80%). In the two blocks, the starting amounts and the probabilities of winning or losing were both fully balanced between the frame conditions. The order of the win and loss block was counterbalanced across participants and other experimental conditions were randomized within the blocks. There was a short break after every 128 trials. Participants were told that their performance in the task determined how much they would be awarded at the end of the experiment. One trial in the win domain (with an initial win amount) and one trial in the loss domain (with an initial loss amount) were randomly chosen and implemented. If the final outcome is losing, participants received no reward. Thus, losing money means winning no money in the end. Because it is not ethic to ask participants to pay out of pocket money in the experiments, randomly choosing one win trial and one loss trial allows participants being motivated to win more and avoid losses. Participants knew they could actually lose money from their base payment and from the money won in the win domain. All the participants received a base payment of 60 yuan (about 10 US dollars) plus any extra reward if they won or lost.

### EEG recording and analysis

EEGs were recorded from 64 scalp sites using Ag/AgCl electrodes embedded in an elastic cap (NeuroScan Inc., USA) according to the international 10–20 system, with the reference to the right mastoid. Eye blinks were recorded from electrodes located above and below the left eye. The horizontal electro-oculogram (EOG) was recorded from electrodes placed 1.5 cm lateral to the left and right external canthi. The EEGs were re-referenced offline to the linked mastoids. All electrode impedances were maintained below 5 kΩ. The EEG and EOG were amplified using a 0.05–70 Hz bandpass and continuously sampled at 500 Hz/channel for off-line analysis.

Ocular artifacts were corrected with an eye-movement correction algorithm using a linear regression approach (Gratton et al., 1983). The data were filtered using a 20 Hz low-pass (24 dB octave roll off), and were baseline corrected by subtracting from each sample the average activity of that channel during the baseline period. For the FRN, a further 1 Hz high-pass filter was used to minimize the influence of P300 on detecting the FRN (Talmi et al., 2012). EEG epochs of 800 ms (with 200 ms pre-stimulus baseline) were extracted off-line for ERPs time-locked to the onset of framing conditions. All trials in which EEG voltages exceeded a threshold of ±70 AV during the recording epoch were excluded from analysis.

According to visual inspection of ERP waveforms, the FRN was measured as the mean amplitudes in the time window of 200–300 ms post-onset of the sure option of different frames. The P300 was measured as the mean value in the 300–500 ms time window on each electrode. We focused on the FRN responses on the anterior frontal midline electrode Fz and the P300 responses on the posterior midline electrode Pz, since the FRN effects and the P300 effects were the largest on these electrodes, respectively. The Mauchly test assesses the validity of the sphericity assumption. Greenhouse-Geisser corrections were used when sphericity was violated. Alpha level for all tests was 0.05. The procedure used in Experiment 2 was similar to Experiment 1 except that the four experimental conditions (win_positive, win_negative, loss_positive, and loss_negative) were fully randomized rather than separated into different blocks. We also used smaller number of trials in Experiment 2 (256 trials in total) than in Experiment 1 (512 trials in total), with 128 win trials and 128 loss trials.

## Results

### Behavioral framing effect

**Figure 2:**
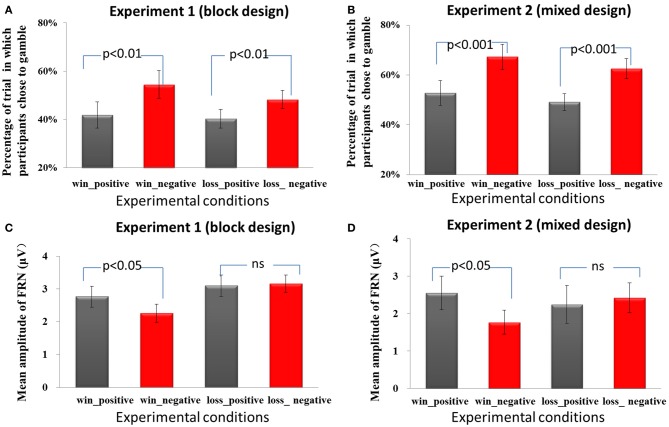
**Behavioral and ERP results**. In Experiment 1 **(A)** and Experiment 2 **(B)**, the frequency of gamble choices (mean, SE) is significant higher in negative frame than in the positive frame, regardless of domain. In both Experiment 1 **(C)** and Experiment 2 **(D)**, the FRN was more negative in the negative frame than in the positive frame only in the win domain but not in the loss domain.

A Two Way repeated measures ANOVA using the domain (win/loss) and the frame (positive/negative) as independent factors and the percentage of gambling choice as dependent factor was performed to analyze the data. As expected, in Experiment 1, using block design, participants revealed a preference for gamble choices in the negative frame compared to the positive frame, *F*_(1, 17)_ = 12.1, *p* = 0.003(see Figure 2A). There was no significant difference between the win and loss domain, *F*_(1, 17)_ = 1.7, *p* = 0.213. The interaction effect was not significant, *F*_(1, 17)_ = 2.6, *p* = 0.123. Furthermore, no significant effects were found for RTs [for the main effect of frame, *F*_(1, 17)_ = 0.1, *p* = 0.771; for the main effect of domain, *F*_(1, 17)_ = 0.5, *p* = 0.502; for the interaction effect, *F*_(1, 17)_ = 0.9, *p* = 0.370]. In addition, the starting amount (￥25, ￥50, ￥75, ￥100) together with the behavioral results for both frame conditions were included as factors in a 4 × 2 analysis of variance (ANOVA) in the win domain and the loss domain separately. A similar ANOVA was performed for the different percentages of the amount offered (20, 40, 60, 80%). No effect of initial reward magnitude or reward probability in the gambling options was found, *p* > 0.05.

In Experiment 2, using a mixed design, we found similar behavioral patterns. The frequency of gamble choices was significantly higher in the negative frame than in the positive frame [*F*_(1, 13)_ = 75.2, *p* < 0.001] (see Figure 2B). There was no significant difference between win and loss domain, *F*_(1, 13)_ = 0.5, *p* = 0.486. The interaction effect was also not significant, *F*_(1, 13)_ = 0.2, *p* = 0.705. The reaction times were not affected by frame conditions, *F*_(1, 13)_ = 0.1, *p* = 0.797. The main effect of domain was marginally significant, *F*_(1, 13)_ = 3.4, *p* = 0.087. The RTs for decisions in the win domain (mean ± *SE*, 722.5 ms ± 52.9) were faster than the RTs in the loss domain (mean ± *SE*, 766.6 ms ± 62.2). The interaction effect of RT was not significant, *F*_(1, 13)_ = 0.03, *p* = 0.854. No effect of initial reward magnitude or reward probability in the gambling options was found, *p* > 0.05.

### FRN effect (1–20 Hz band-pass)

**Figure 3:**
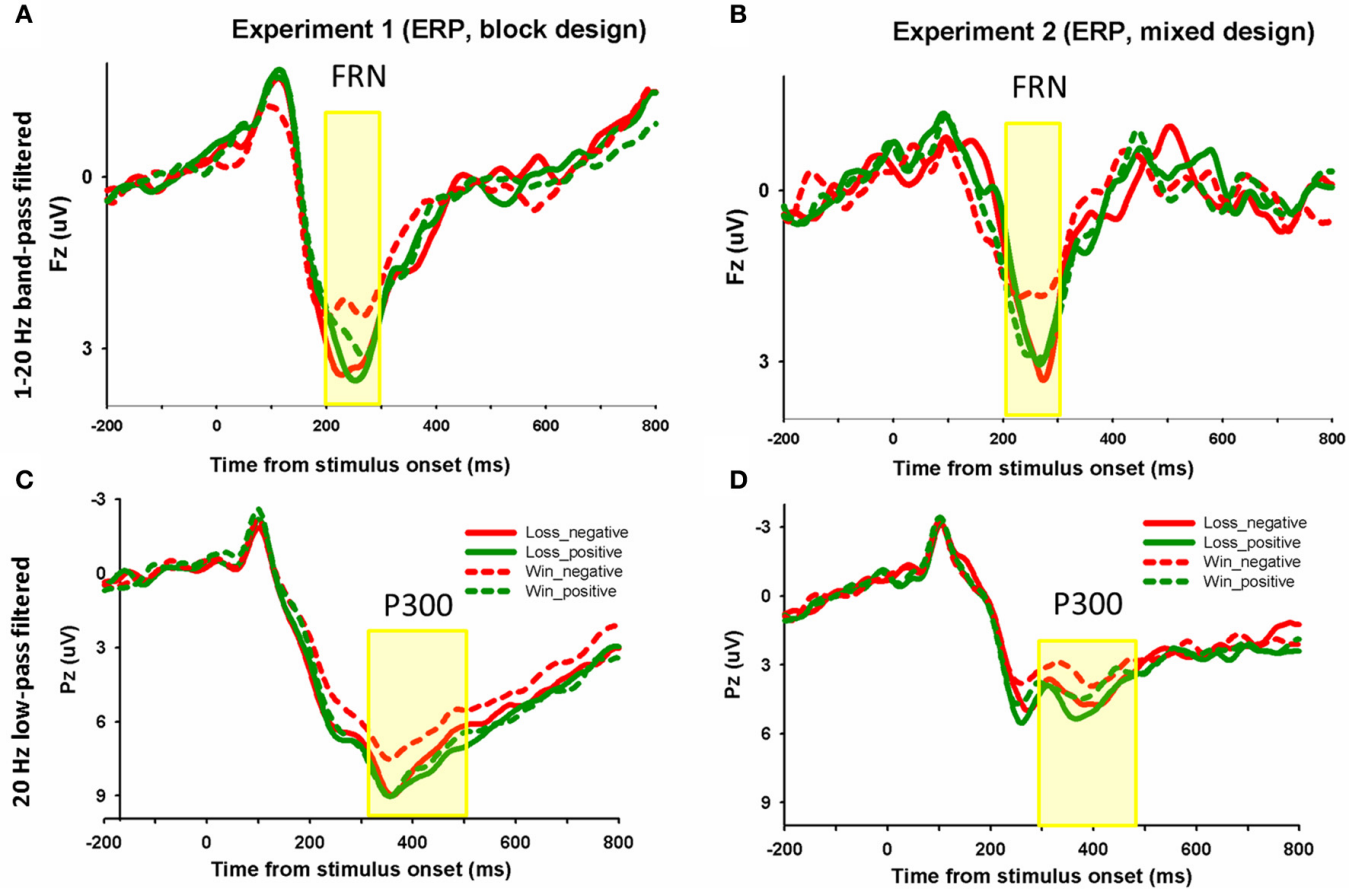
**The ERP grand-average waveforms**. In Experiment 1 **(A)** and Experiment 2 **(B)**, grand-average waveforms from channel FZ using 1–20 Hz band-pass filtered for four experimental conditions (win_positive, win_negative, loss_positive, loss_negative) were shown. Similar waveforms were plotted for ERP at channel PZ using 20 Hz low-pass filtered **(C,D)**. The shaded 200–300 and 300–500 ms time window were used to measure the FRN and P300 magnitude, respectively.

Each of the 18 participants in Experiment 1 had at least 120 trials and each of the 14 participants in Experiment 2 had 60 trials in each condition for EEG averaging. The group waveforms for 4 experimental conditions after 1–20 Hz band-pass filtering were plotted in Figures 3A,B. we performed a repeated-measures ANOVA with the factors domain (win or loss) and frame (positive or negative). The mean amplitude of FRN on the electrode FZ in the 200–300 ms time window and the mean values of P300 on the electrode PZ in the 300–500 ms time window were entered into repeated ANOVA analysis. The main effect of domain on the FRN was significant, *F*_(1, 17)_ = 4.5, *p* = 0.048, FRNs in the win block were more negative (mean ± *SE*, 2.5 μ V ± 0.3) than those in the loss block (mean ± *SE*, 3.1 μ V ± 0.3). The main effect of frame was not significant, *F*_(1, 17)_ = 1.6, *p* = 0.228. The interaction effect was significant, *F*_(1, 17)_ = 4.5, *p* = 0.049. Pairwise *t*-tests on simple effects showed that in the win block, FRNs in the negative frame (mean ± *SE*, 2.3 μ V ± 0.3) were more negative than FRNs in the positive frame (mean ± *SE*, 2.8 μ V ± 0.3), *t*_(17)_ = 2.2, *p* = 0.04. However, there was no significant difference between the positive and negative frames in the loss domain, *t*_(17)_ = −0.3, *p* = 0.766 (see Figures 2C, 3A). These results suggest that the positive/negative framing effect on FRN only exists in the win domain. Moreover, in order to control for the possible effect of magnitude on the in the observed frame × domain interaction, an ANOVA with domain (win or loss), frame (positive or negative) and magnitude (low [￥25, ￥50] or high [￥75, ￥100]) as independent factors revealed a significant main effect of domain, *F*_(1, 17)_ = 4.5, *p* = 0.048 and a significant interaction effect between domain and frame, *F*_(1, 17)_ = 4.5, *p* = 0.048. No other effects were significant, *p* > 0.1.

In Experiment 2, the FRN results revealed a significant framing × domain interaction effect, *F*_(1, 13)_ = 4.9, *p* = 0.046. The main effect of framing or domain was not significant, *F*_(1, 13)_ = 2.0, *p* = 0178, *F*_(1, 13)_ = 0.4, *p* = 0.538, respectively. Pairwise *t*-tests on simple effects suggested that in the win domain, FRN being more negative to the negative frame (mean ± *SE*, 1.8 μ V ± 0.3) than to the positive frame (mean ± *SE*, 2.6 μ V ± 0.5), *t*_(13)_ = 2.3, *p* = 0.039. However, in the loss domain, there was no significant difference between the two types of frames, *t*_(13)_ = 0.7, *p* = 0.515 (see Figures 2D, 3B). Since there were not enough trials (less than 20) to analyze the data to investigate the effect of magnitude, we did not add the magnitude factor to analyze the data.

**Figure 4:**
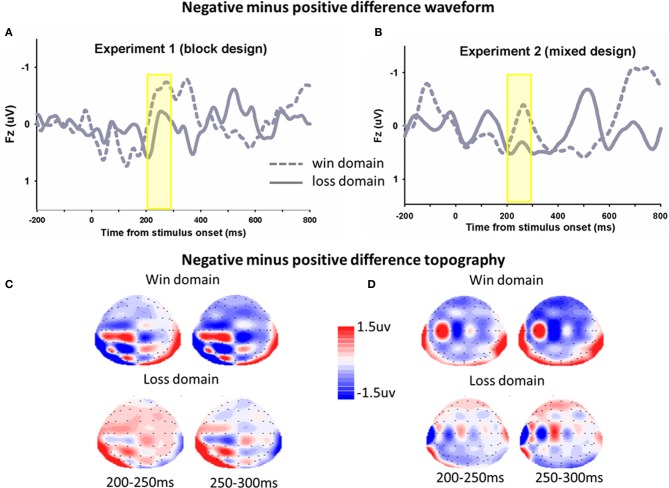
**The ERP difference waveforms and topograph (FRN)**. In Experiment 1 **(A)** and Experiment 2 **(B)**, difference waveforms (negative minus positive) in the win domain and the loss domain were shown. The corresponding topographical maps (200–300, 50 ms increment) were shown **(C,D)**.

The same analyses were conducted for the average magnitude of FRN on the electrode CZ. In Experiment 1, The main effect of domain was significant, *F*_(1, 17)_ = 4.9, *p* = 0.041, FRNs in the win block were more negative (mean ± *SE*, 2.0 μ V ± 0.4) than those in the loss block (mean ± *SE*, 2.6 μ V ± 0.3). The main effect of frame was not significant, *F*_(1, 17)_ = 1.0, *p* = 0.323. The framing × domain interaction effect was significant, *F*_(1, 17)_ = 8.0, *p* = 0.011. Pairwise *t*-tests on simple effects showed that in the win block, FRNs in the negative frame (mean ± *SE*, 2.2 μ V ± 0.4) were more negative than FRNs in the positive frame (mean ± *SE*, 1.8 μ V ± 0.4), *t*_(17)_ = 2.2, *p* = 0.04. However, there was no significant difference between the positive and negative frames in the loss domain, *t*_(17)_ = −0.4, *p* = 0.690. In Experiment 2, there was no significant main effect of domain *F*_(1, 13)_ = 0.03, *p* = 0.866, and no significant interaction effect, *F*_(1, 13)_ = 2.1, *p* = 0.169. The main effect of frame approached significant, *F*_(1, 13)_ = 3.9, *p* = 0.089. The difference waveforms and corresponding topographical maps were shown in Figure 4.

The measurement of the FRN may be confounded by the P300 effect. To deal with this issue, we measured the P300 after using the same 1–20 Hz band-pass filtering. Since the FRN is consisted of bursts of activity in the 4–7 Hz frequency range (Willoughby and Gehring, 2004; Cohen et al., 2007) and the long-duration component P300 is in the frequency range (<3 Hz) (Ford et al., 2008), high-pass filtering (1 Hz) can minimize the P300 effect while preserve the FRN effect. Here, the P300 was measured as the mean amplitude of 300–500 ms time window. In Experiment 1, the main effect of domain on P300 was not significant, *P* > 0.05. The main effect of domain and the interaction effect were not significant (*p* values > 0.3). In Experiment 2, the main effect of frame, the main effect of domain, and the interaction effect were all not significant (*p* values > 0.1). Thus, the P300 showed different patterns from the FRN. These results suggest that the observed FRN effect in the present experiments cannot simply be explained by the P300 effect. No significant correlation was found between the FRN effect and the behavioral framing effect, possibly due to the small sample size.

### P300 effect (20 Hz low-pass)

The group waveforms for 4 experimental conditions after 20 Hz low-pass filtering were plotted in Figures 3C,D. In Experiment 1, the main effect of frame on P300 was significant, *F*_(1, 17)_ = 10.5, *p* = 0.005. The P300 was more positive in the positive frame (mean ± *SE*, 7.9 μ V ± 0.6) than in the negative frame (mean ± *SE*, 7.1 μ V ± 0.7). The main effect of domain was not significant, *F*_(1, 17)_ = 1.6, *p* = 0.221. The interaction effect was not significant, *F*_(1, 17)_ = 2.5, *p* = 0.13.

**Figure 5:**
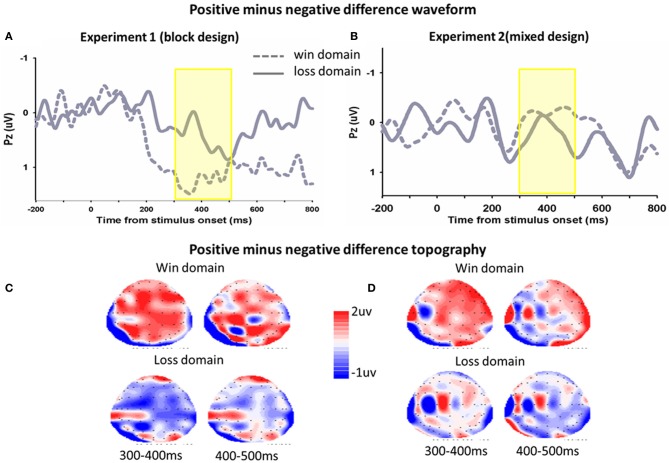
**The ERP difference waveforms and topograph (P300)**. In Experiment 1 **(A)** and Experiment 2 **(B)**, difference waveforms (positive minus negative) in the win domain and the loss domain were shown. The corresponding topographical maps (300–500 ms, 100 ms increment) were shown **(C,D)**.

In Experiment 2, the main effect of frame on P300 was marginally significant, *F*_(1, 13)_ = 4.0, *p* = 0.067. The P300 was more positive in the positive frame (mean ± *SE*, 4.1 μ V ± 1.2) than in the negative frame (mean ± *SE*, 3.7 μ V ± 1.3). The main effect of domain was also marginally significant, *F*_(1, 13)_ = 3.4, *p* = 0.088. The P300 in the loss domain (mean ± *SE*, 4.2 μ V ± 1.3) was more positive than that in the win domain (mean ± *SE*, 3.6 μ V ± 1.3). The interaction effect was not significant, *F*_(1, 13)_ < 1. The difference waveforms and corresponding topographical maps were shown in Figure 5.

## Discussion

Across two experiments, we demonstrate robust framing effect in both the win and the loss domains. Framing influences choices in both domains: subjects are more risk averse in the positive frame and more risk seeking in the negative frame, both in the domain of wins and losses. Importantly, our ERP studies show that the initial evaluations of the positive and the negative frame differ within 300 ms in the win domain. However, in both ERP studies, we did not find significant ERP difference between the two differently valenced frames in the loss domain, even though behavioral framing effects were significant.

Our results lend some neural support to the prospect theory accounts of framing effect which posits that positive frames are encoded as gains whereas negative frames are encoded as losses, suggesting that the description invariance principle is indeed violated in the framing paradigm. Two descriptions, “keep ￥60” and “lose ￥40” when the initial amount is ￥100, represented the identical option, but elicited distinct FRN and P300. The FRN was more negative for “lose ￥40” than for “keep ￥60” even though participants can easily reason that losing ￥40 out of ￥100 equals keeping ￥60. According to the motivational accounts of the FRN which suggest that the FRN is sensitive to the valence of outcomes (Gehring and Willoughby, 2002), our data indicate that negative frames are encoded as losses whereas positive frames are encoded as gains in the win domain. The FRN effect cannot simply be explained by the valence of the words we used. For example, both words “keep” and “save” are positive but they elicited distinct FRNs. Moreover, previous studies using pleasant and unpleasant affectively valent words did not find differences in the FRNs in the time window between 200 and 300 ms post-stimuli (Kiehl et al., 1999; Bernat et al., 2001). Our results on P300 are also consistent with the view that the P300 is related to processes of attentional allocation and to high-level motivational/affective evaluation, being more positive for more positive stimuli (Olofsson et al., 2008). Thus, we provide direct neural evidence that the description invariance principle is violated and outcome evaluation depends on its representation. Our data favor the prospect theory account of framing to other theories that require deliberate reasoning.

It is surprising that the frame effect on FRN was only significant in the win domain but not in the loss domain. One possibility is that the negative frame in the win domain is encoded as “worse than expected” negative prediction error, whereas the positive frame in the loss domain is encoded as “better than expected” positive prediction error, and the FRN is only sensitive to negative prediction error (Nieuwenhuis et al., 2004). It is possible that when the initial amount is a gain, “lose ￥40” is encoded as a loss and produces a “worse than expected” negative prediction error. Previous studies have consistently shown that the FRN is sensitive to the negative prediction error (Holroyd et al., 2003, 2008, 2009; Holroyd and Krigolson, 2007). However, findings are mixed regarding whether the FRN is sensitive to the positive prediction error as well. Some studies have shown that the FRN is sensitive to the positive prediction error but with much smaller magnitude, compared with its sensitivity to the negative prediction error (Oliveira et al., 2007; Yu et al., 2011). Some studies found no effects of the positive prediction error on FRN amplitude (Holroyd et al., 2003; Krigolson and Holroyd, 2007; Bellebaum et al., 2010). It remains unclear why the ACC (or FRN amplitude) appears to be less responsive to the positive prediction error. A number of recent studies suggest that dopamine and serotonin neuromodulators contribute differentially to coding for outcomes in the win and the loss domain, respectively. For example, it has been shown that dopamine agonists affected choices in the gain domain (both neurally and behaviorally) but not the loss domain (Pessiglione et al., 2006). Genetic variation in tonic dopamine and serotonin levels modifies risk seeking in gain and loss domains, respectively (Zhong et al., 2009). Accordingly, given that the FRN is believed to reflect a dopaminergic signal, we should not be surprised to see that it only reflects negative prediction error. Recent ERP studies also showed no significant difference in the FRN for good and bad outcomes in the loss domain (Kreussel et al., 2012; Sambrook et al., 2012). A recent study demonstrated that size and probability of rewards modulate the FRN associated with wins but not losses (San Martin et al., 2010). This might be due to a separation of dopamine and serotonin coding functions in gain and loss domains. It is also possible that different sub-regions in dopaminergic midbrain and the striatum encode different types of prediction error and positive prediction error may not be sent to the ACC (Bayer and Glimcher, 2005; Pessiglione et al., 2006; Cohen et al., 2010). Our results contribute to a growing body of empirical evidence showing a greater modulation of the FRN for win feedback in comparison to loss feedback (Cohen et al., 2007; Holroyd et al., 2008; San Martin et al., 2010). Our findings, if replicated, suggest that different neural substrates may be involved in modulating framing effect in the win and the loss domain. Although previous neuroimaging studies focus on the framing effect in the win domain (De Martino et al., 2006; Roiser et al., 2009), the neural correlate of the framing effect in the loss domain is still unclear. There is accumulating evidence suggesting that the neural mechanisms underlying win and loss processing are different (O'Doherty et al., 2001, 2003, 2006; Ullsperger and von Cramon, 2003; Kringelbach, 2005; Nieuwenhuis et al., 2005; Liu et al., 2007). Other neuroimaging methods (e.g., fMRI) are needed to further examine the neural basis of framing in the loss domain.

Previous neuroimaging studies on framing only focus on the decision stage. Using the similar economic decision-making paradigm, two studies compared choices in accordance with the framing effect and choices against the framing effect at the decision stage (De Martino et al., 2006; Roiser et al., 2009). These studies highlight the interplay between prefrontal cortex and amygdala in framing effect. The ACC is interpreted as exerting cognitive control over emotional response in amygdala. It has been shown that choices made counter to, relative to those made in accord with, the frame were associated with increased anterior cingulate–amygdala coupling in individuals with homozygous for the long (la) allele at the 5-HTTLPR (Roiser et al., 2009). However, amygdala lesion patients did not show abnormal framing effect (Talmi et al., 2010), suggesting that the amygdala may not play a causal role in framing, although it contributes to decision making in framing. Our findings suggest that the ACC may not only contribute to framing effect by inhibiting amygdalar activity, but is also involved in the motivational evaluation of stimuli. Taken together, our findings suggest an important role of the ACC in framing.

Another two fMRI studies on framing effect used the Asian disease problem. One study compared risky choices with sure option choices and found that the cognitive effort required to select a sure gain was considerably lower than that required to choose a risky gain in the positive frame but not in the negative frame (Gonzalez et al., 2005). Activation in frontal, parietal areas differed between risky and certain choices, but only for the positive and not for the negative frame. Another study compared choices in the positive frame with choices in the negative frame and found that choices in the positive frame were associated with enhanced activity in inferior frontal gyrus, insula and parietal lobe (Zheng et al., 2010). No significantly increased neural activity for choices in the negative frame was reported. Our findings extend these studies by showing differential encoding of frame before decisions are made.

It is worth noting that both behavioral and neural responses to frame are different between the blocked design experiment (Experiment 1) and the mixed design experiment (Experiment 2), suggesting that our experimental manipulation did influence subjects' behavioral and brain responses. In Experiment 2, as Figure 2 shows, participants were generally more likely to gamble across conditions, compared with their probability of gambling in Experiment 1. When wins and losses were presented within a block, the contrast between wins and losses became salient. This may induce a general loss aversion tendency and the risk-seeking strategy to compensate loss aversion (i.e., gamble in the hope to keep all wins and avoid all losses). Moreover, in Experiment 2, the RTs for decisions in the win domain tended to be faster than the RTs in the loss domain (*p* = 0.087), in contrast with findings that there was no RT difference between domains in Experiment 1. It is possible that when win and loss domains are mixed, the switching between domains makes making decisions about losses more difficult. For the ERP results, as Figures 2, 3 shows, the ERP amplitudes were overall smaller in Experiment 2 than in Exp eriment 1. The results may be attributable to individual differences in these relatively small samples. Nevertheless, the main effects of frames remain significant and consistent across experiments, suggesting that our behavioral and ERP results are stable. It is important to notice that the loss-associated FRNs were not more negative than win-associated FRNs in the current study probably because that participants already received win/loss feedback in the “initial amounts” stage. Thus, information in the “sure amounts” stage does not provide additional information on the win/loss dimension. The FRN may respond more to the framing manipulation rather than to the already known win/loss dimension. Moreover, the ERP waveforms in Figures 2C,D show that in the loss domain, both negative and positive frames were coded as gains (i.e., they appear to be comparable in magnitude to the “win_positive” condition and the “win_negative” condition). In other words, when starting with an initial loss, it appears that both frames are coded as relatively advantageous outcomes. The ERP response here might be understood as the reward positivity to predictive cues (Holroyd et al., 2011). While FRN amplitude did not vary by frame valence in the loss domain, the fact that these two conditions appear to be coded as gains might be potentially meaningful. Future studies may further investigate this phenomenon.

Some limitations in our study are worth mentioning. First, although the ACC is generally believed to be the main generator of the FRN (Miltner et al., 1997; Gehring and Willoughby, 2002; Nieuwenhuis et al., 2004; Martin et al., 2009), our ERP studies did not provide direct evidence to link FRN amplitude with ACC activity. Other neuroimaging methods with high spatial resolution are needed to locate the source of FRN more precisely. Although it is widely believed that the FRN is generated in the ACC, recent studies show that the sources of the FRN might be widely distributed (Carlson et al., 2011; Foti et al., 2011). Second, although we provide evidence that framing influences the initial option evaluation processes, it is still unclear whether framing also influences the subsequent decision-making processes. The interactions among several brain regions may underlie the effects of framing at the decision stages, and these processes could be better examined using fMRI. Third, due to the poor spatial resolution of ERP, our studies are silent about the brain regions underlying the observed framing effects in the win and loss domains. Finally, the P300 results were inconsistent across the two experiments in our study, possibly due to the difference in the experimental design (blocked vs. mixed). Although not the focus of the current study, we also reported the P300 findings the sake of completeness. The functional significance of the P300 in reward processing is still under debate. More studies are needed to further elucidate the role of the P300 in assessing outcomes.

People make judgments based on their representations of events, rather than on the events themselves. Decision making is not description-invariant, as would be expected on a normative theory, and hence can change according to the representation that is provided. In prospect theory, it is the decision maker's private framing of the problem in terms of gains or losses that determines her evaluation of the options. Our findings demonstrate that framing influences decisions in both the win and the loss domains and provide neural evidence that the description invariance principle is violated in the framing effect.

## Author contributions

Rongjun Yu conceived the study. Ping Zhang analyzed the data. Rongjun Yu wrote the paper. We thank Li Li for collecting the data. All authors edited the manuscript. All authors read and approved the final manuscript.

### Conflict of interest statement

The authors declare that the research was conducted in the absence of any commercial or financial relationships that could be construed as a potential conflict of interest.
